# Magnetic Accumulation of SPIONs under Arterial Flow Conditions: Effect of Serum and Red Blood Cells

**DOI:** 10.3390/molecules24142588

**Published:** 2019-07-16

**Authors:** Till L. Hennig, Harald Unterweger, Stefan Lyer, Christoph Alexiou, Iwona Cicha

**Affiliations:** Section of Experimental Oncology und Nanomedicine (SEON), ENT-Department, Universitätsklinikum Erlangen, Friedrich-Alexander Universität Erlangen-Nürnberg, 91054 Erlangen, Germany

**Keywords:** SPIONs, magnetic targeting, nanoparticle accumulation, ex vivo flow model, blood cells

## Abstract

Magnetic drug targeting utilizes an external magnetic field to target superparamagnetic iron oxide nanoparticles (SPIONs) and their cargo to the diseased vasculature regions. In the arteries, the flow conditions affect the behavior of magnetic particles and the efficacy of their accumulation. In order to estimate the magnetic capture of SPIONs in more physiological-like settings, we previously established an ex vivo model based on human umbilical cord arteries. The artery model was employed in our present studies in order to analyze the effects of the blood components on the efficacy of magnetic targeting, utilizing 2 types of SPIONs with different physicochemical characteristics. In the presence of freshly isolated human plasma or whole blood, a strong increase in iron content measured by AES was observed for both particle types along the artery wall, in parallel with clotting activation due to endogenous thrombin generation in plasma. Subsequent studies therefore utilized SPION suspensions in serum and washed red blood cells (RBCs) at hematocrit 50%. Interestingly, in contrast to cell culture medium suspensions, magnetic accumulation of circulating SPION-3 under the external magnet was achieved in the presence of RBCs. Taken together, our data shows that the presence of blood components affects, but does not prevent, the magnetic accumulation of circulating SPIONs.

## 1. Introduction

Nanomedicine offers a unique platform for novel drug delivery approaches to the therapy of cardiovascular diseases (CVD). Thus far however, clinical impact of nanomedicine in diagnosis or therapy of CVD has been scarce, despite intensive research efforts [1,2]. One reason for this is an insufficient accumulation of nanoparticles in the diseased arteries [3]. To achieve the required efficacy, the parenterally-administered particles should have a long circulation half-life and a high margination rate, and allow enhanced interactions with endothelial cells in the target vasculature. In arterial circulation, shear stress-activated processes may significantly affect the nanoparticle internalization by endothelial cells. Moreover, due to the larger size of the vessels, the rheological behavior of blood cells in the arterial flow differs from that in the microvessels [4,5,6]. Red blood cell (RBC) accumulation in the center of the lumen and the formation of rouleaux, creates a cell-free layer at the vessel wall, which may strongly affect the margination of nano-sized particles [7]. Previous studies showed that in flowing blood suspensions, platelets [8] and platelet-sized beads with 2.38 μm diameter [9] strongly accumulate within the cell-free layer near the vessel walls, as compared with the central region of the flow. According to the mathematical model developed by Lee et al., this is due to the fact that ellipsoidal microparticles display stronger hydrodynamic margination under flow than sub-micrometer and nanometer particles [10]. From this model, the minimum equivalent radii for observing margination under normal hemodynamic conditions (i.e., shear rate of 10^3^/s) with no external forces would be 7 µm for silica, or 3.5 µm for iron oxide particles. For particles with nanometer size-range, the modelled contributions of the inertial and gravitational forces under physiological conditions are negligibly small, preventing their margination [10]. These mathematical predictions indicate that, in the absence of external forces, sub-micrometer and nanometer particles of any shape within the circulation can only oscillate around their trajectory. Passive targeting can thus be used to deliver nanotherapeutics to the diseased regions, but its efficacy is limited to the vascular beds where the blood–tissue barrier in horizontal capillaries is compromised. Conjugating nanoparticles to specific ligands that target endothelial activation markers is considered a useful approach to enhance the internalization of particles under arterial flow. However a series of comprehensive ex vivo investigations concerning the endothelial interactions with nano- and microparticles conjugated with sialyl Lewis^a^ (sLe^a^), a ligand specific to the endothelial selectins, showed that nanoparticles (100–500 nm) displayed minimal margination towards the endothelial monolayer in human whole blood flowing in rectangular chambers of varying heights [11,12]. In contrast, a significantly higher binding of intermediately-sized microspheres (2–5 µm) was detected in this model [11]. Notably, whereas microparticle attachment to the endothelium was 2 to 4-fold increased under pulsatile blood flow compared to laminar flow, the margination of nanoparticles was not enhanced, and although margination of rod-shaped microparticles with high aspect ratios was significantly improved as compared to spheres of equal volume, the nanorods did not display enhanced margination compared to that of nanospheres [12]. The authors concluded that both nanorods and nanospheres show no margination in the presence of RBCs in vitro, confirming the theoretical predictions of Lee et al. [10]. These in vitro and ex vivo results indicate that many types of nanocarriers may not be adequate for vascular applications in human arteries due to their small size and/or insufficient margination from the bloodstream. Extensive efforts are therefore focused on identifying efficient targeting approaches that could enhance the binding of nanoparticles to vascular endothelium at the disease-specific regions, including medium and large vessels.

One of these approaches, so-called magnetic drug targeting (MDT) utilizes an external magnetic field to accumulate superparamagnetic iron oxide nanoparticles (SPIONs) and their cargo at the diseased vasculature regions [13]. The magnetic properties of SPIONs allow their visualization by magnetic resonance imaging, but also the remote control of their accumulation in vivo. However, in contrast to their broad utility as imaging agents [14,15,16,17,18], relatively few reports addressed the use of SPIONs for cardiovascular drug delivery thus far [19,20,21]. In spite of the fact that a number of papers were previously published concerning magnetic targeting of SPIONs under flow conditions, the majority of studies [22,23,24,25] addressed this process either in flow models that do not reflect arterial wall geometry (glass or plastic tubes) or in cancer models, where due to increased endothelial permeability and EPR effect, the leakage of magnetofluids in the tissue is easily achieved. Magnetic capture of nanoparticles under flow conditions characteristic for larger vessels may be more difficult to achieve, as the force exerted by the magnetic field must overcome the hydrodynamic (drag) force and the shear effects generated by the flowing blood cells. Apart from the specific nanoparticle characteristics [26], the magnetic field gradients and the flow dynamics thus determine the behavior of magnetic particles in circulation and the efficacy of their accumulation. In order to estimate the magnetic capture of SPIONs in more physiological-like settings, we previously established an ex vivo model based on the human umbilical cord arteries [27]. Using this model, we investigated the magnetic targeting of SPIONs suspended in cell culture media under various external magnetic field gradients and flow conditions, showing that accumulation of some types of SPIONs at the arterial wall is achievable under the guidance of a sufficiently strong external magnet. As only cell culture media were used in the previous study, among the unanswered questions are the effects of blood components (both at the cellular and protein level) on nanoparticle behavior under arterial flow conditions. It is extremely difficult to estimate these effects based on mathematical simulations, and thus far comprehensive standardized studies are missing that would investigate the efficacy of particle accumulation under external magnetic field in the presence of blood components. To address these questions, the artery model was employed in our present studies in order to analyze the effects of the blood components on the efficacy of magnetic targeting, utilizing SPIONs with different physicochemical characteristics. The novelty of this work involves the use of human tissue model to investigate the possibility and the conditions required for capture of circulating SPIONs under uninterrupted arterial flow conditions in the presence of blood components.

## 2. Results

**Figure A1:**
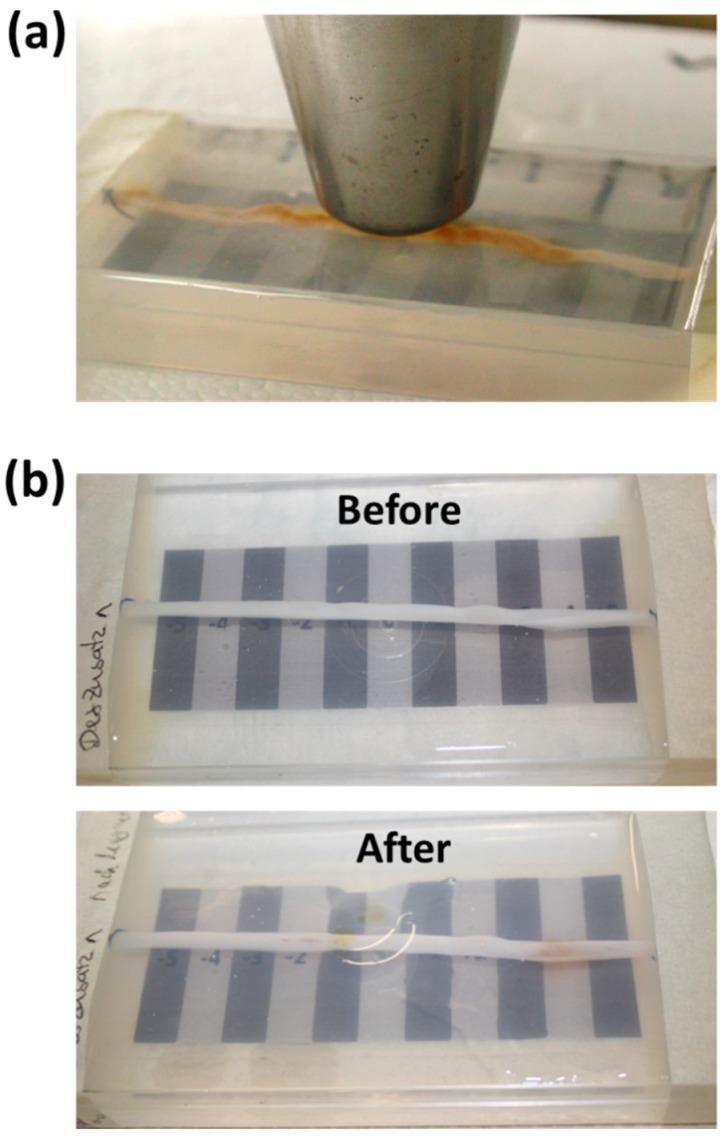
Ex vivo umbilical artery model: (**a**) Placement of the magnet tip; (**b**) representative macroscopic images of the artery model before and after the flow experiment.

To answer the need for an easy to handle basic research model system for MDT investigations under arterial flow conditions, we previously developed and reported an ex vivo model based on the branch-free human umbilical cord arteries (Figure A1). Originally, three types of SPIONs were considered, including lauric acid-coated iron oxide nanoparticles synthesized according to Khalafalla et al. [28] (SPION-1, with 126 nm hydrodynamic diameter and ζ-potential of –34.6 mV), as well as SPION-2 with improved lauric acid/albumin coating and SPION-3 with crosslinked dextran coating. In our present study, we focused on investigations of SPION-2 and SPION-3, due to the fact that SPION-1 showed limited stability in human blood and poor batch to batch reproducibility. For the sake of consistency, we kept the original terminology of the tested SPIONs. The detailed characterization of these particles was reported before and is summarized in Supplementary Table S1 and Supplementary Figure S1.

### 2.1. Magnetic Capture Efficacy of SPIONs in the Presence of Plasma and Whole Blood

In our present study, the ex vivo artery model was used to evaluate the magnetic accumulation of SPIONs with different coatings and physicochemical properties in the presence of blood components. The following particles were used: SPION-2 with lauric acid/albumin shell (71 nm hydrodynamic diameter and ζ-potential of −18.3 mV), and SPION-3 with dextran shell (74 nm hydrodynamic diameter and ζ-potential of −3.8 mV). Both these particle types have been shown to be stable in whole blood (Supplementary Figure S2) [29,30]. We never observed any agglomeration in SPION-2 or SPION-3 samples in the presence of serum proteins, nor the induction of plasma coagulation (Supplementary Figure S3). Due to their good biocompatibility, these SPIONs are currently used in proof-of-feasibility animal studies for different clinical applications [21,29].

**Figure 1:**
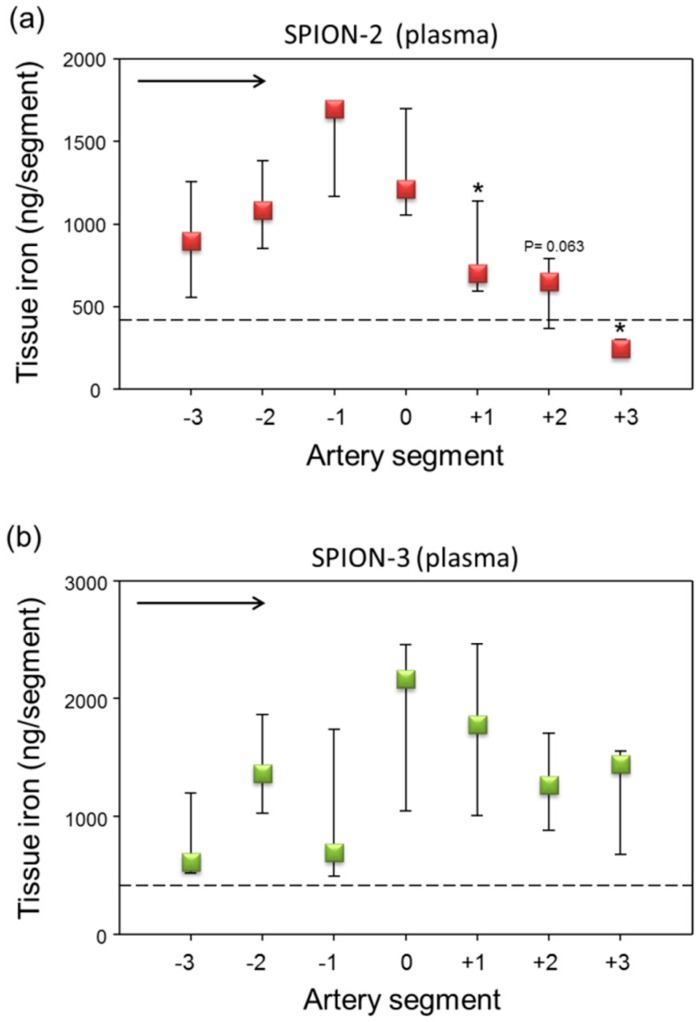
Magnetic targeting of circulating superparamagnetic iron oxide nanoparticles (SPION)-2 and SPION-3 suspended in plasma. (**a**) SPION-2 and (**b**) SPION-3 suspensions at 30 µg Fe/mL were circulated in the model for 30 min under external magnetic force positioned at segment 0. Arrow indicates the direction of flow. Shown is iron concentration of respective segments (median with 75th and 25th percentile of *n* = 5 experiments). Average control values (SPIONs suspensions without magnet) are indicated by dashed line. * *P* < 0.05 versus segment 0; trend *P* values are indicated.

In the first set of experiments, we prepared the respective SPION suspensions using freshly isolated human plasma or whole blood. In plasma, a strong peak in SPION-2 accumulation under the magnet, slightly shifted towards segment −1 was observed (Figure 1a). The iron content measurements in SPION-3 samples also demonstrated a presence of a peak around segments 0 and +1, but there was a large variability between single experiments (Figure 1b). There were no major differences between the SPION-2 and SPION-3 in the absolute iron concentrations (peak levels between 1000–2000 ng Fe/segment).

**Figure 2:**
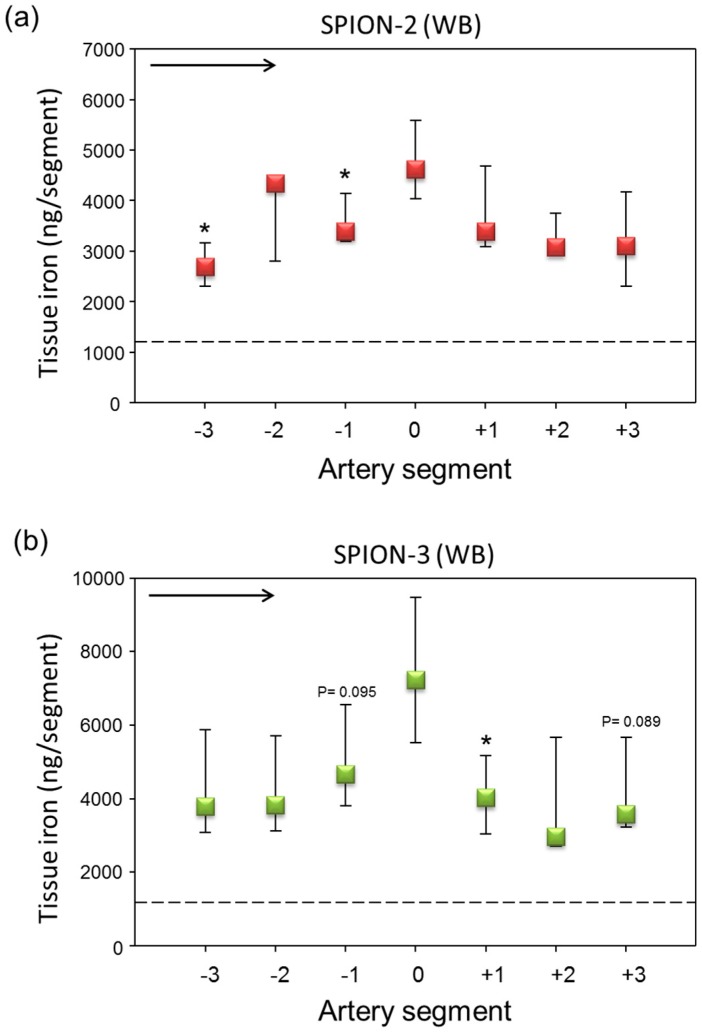
Magnetic targeting of circulating SPION-2 and SPION-3 suspended in whole blood (WB). (**a**) SPION-2 and (**b**) SPION-3 suspensions at 30 µg Fe/mL were circulated in the model for 30 min under external magnetic force positioned at segment 0. Arrow indicates the direction of flow. Shown is iron concentration of respective segments (median with 75th and 25th percentile of *n* = 5 experiments). Average control values (SPIONs suspensions without magnet) are indicated by dashed line. * *P* < 0.05 versus segment 0; trend *P* values are indicated.

**Figure 3:**
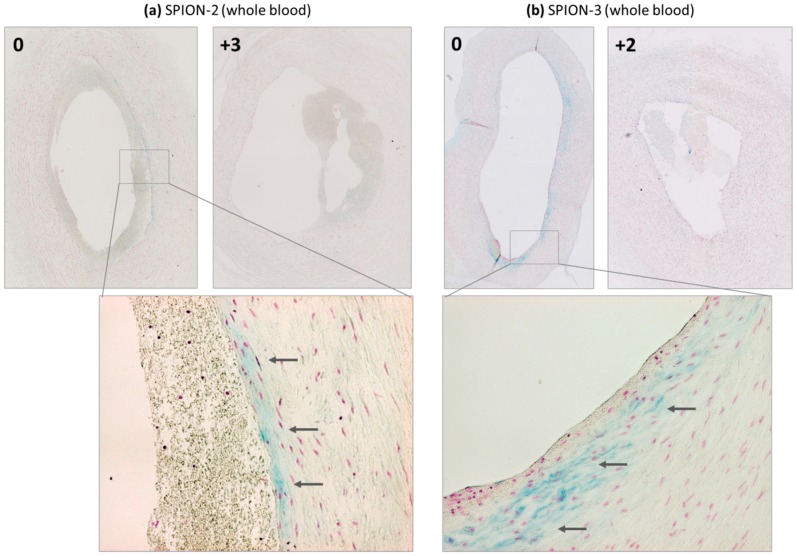
Detection of iron presence in arterial wall by histology. Magnetically targeted (segment 0) and representative control segments away from the tip of the magnet are shown for (**a**) SPION-2 and (**b**) SPION-3, suspended in the whole blood. Arrows indicate blue staining due to the particle accumulation.

When particles were suspended in freshly isolated citrated human blood, an overall strong increase in iron content along the artery wall was measured, which may be related to partial hemolysis of the RBCs during the multiple circulation rounds within the flow system. A mild peak under the magnet (segment 0) was observed for SPION-2 (Figure 2a). Interestingly, a stronger iron accumulation and a more pronounced peak were observed in SPION-3 samples (Figure 2b). Figure 3 shows the representative histological images of the respective segments 0 under the magnetic tip, stained with Prussian blue.

**Figure 4:**
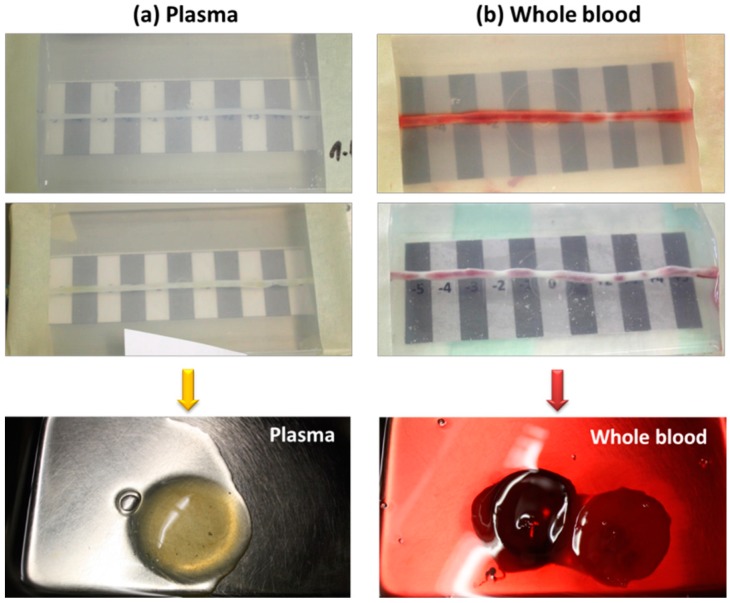
Representative macroscopic images of the artery models after the experiments with (**a**) plasma and (**b**) whole blood. Lower panel shows clots washed out after the experiments.

It must be noted, however, that during perfusion of SPION suspensions either in plasma or in whole blood through the umbilical arteries from different donors, a strong increase in clotting was detected (Figure 4). This efect was not observed in SPION suspensions before the perfusion through the artery model, or in our previous in vitro studies (Supplementary Figures S2 and S3), so the pro-coagulatory state was clearly induced by the activation of endogenous blood thrombin upon contact with arterial vessel wall.

### 2.2. Magnetic Capture of SPIONs in the Presence of Serum and Washed RBC Suspensions

**Figure 5:**
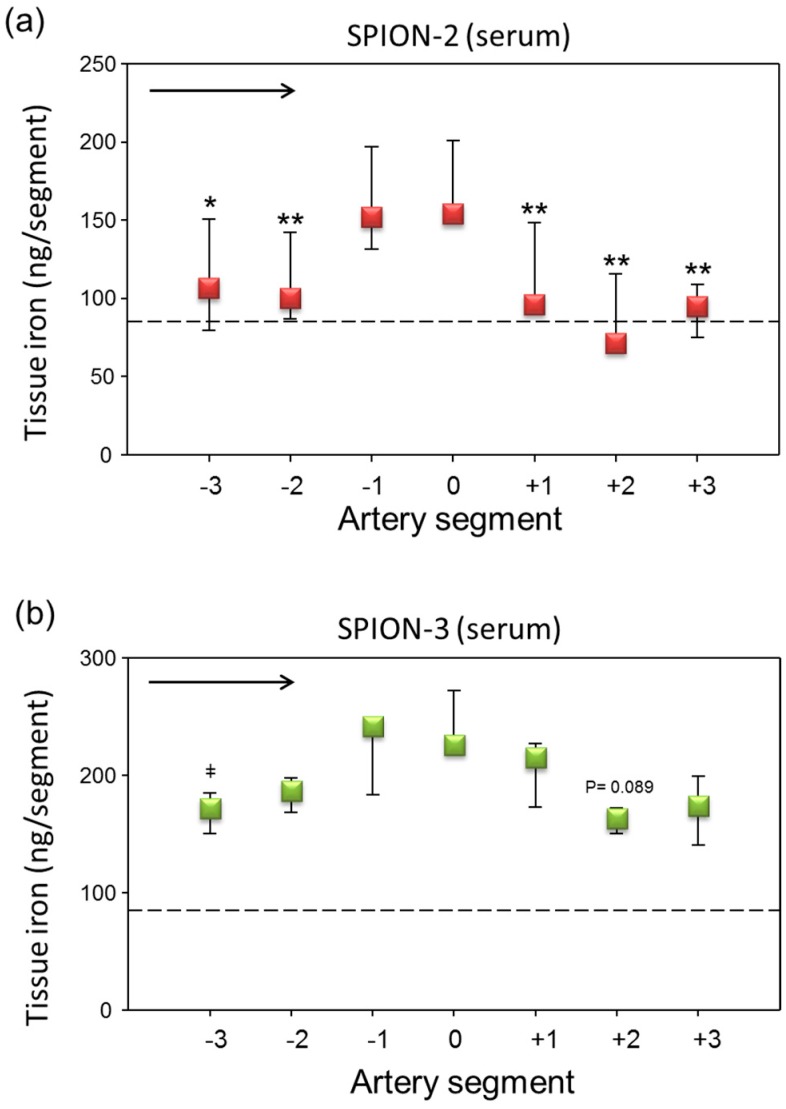
Magnetic targeting of circulating SPION-2 and SPION-3 suspended in serum. (**a**) SPION-2 and (**b**) SPION-3 suspensions at 30 µg Fe/mL were circulated in the model for 30 min under external magnetic force positioned at segment 0. Arrow indicates the direction of flow. Shown is iron concentration of respective segments (median with 75th and 25th percentile of *n* = 5 experiments). Average control values (SPIONs suspensions without magnet) are indicated by dashed line. ** *P* < 0.01, * *P* < 0.05, versus segment 0; ‡ *P* = 0.066 versus segment −1; trend *P* values are indicated.

To overcome the problem of clotting, human serum (cell-free part of blood devoid of clotting factors such as fibrinogen) or washed RBC resuspended in PBS to 50% hematocrit were used in the subsequent set of experiments. Under these conditions, no clotting activation was observed in the perfused samples, either controls or nanoparticle-containing suspensions. Compared to plasma samples, the SPION suspensions in serum showed a very week margination and only mild peaks were observed for both types of SPIONs around segments 0 and −1 (Figure 5). The absolute values of iron measured with AES were very low, around 150–220 ng Fe/segment in the peak regions.

**Figure 6:**
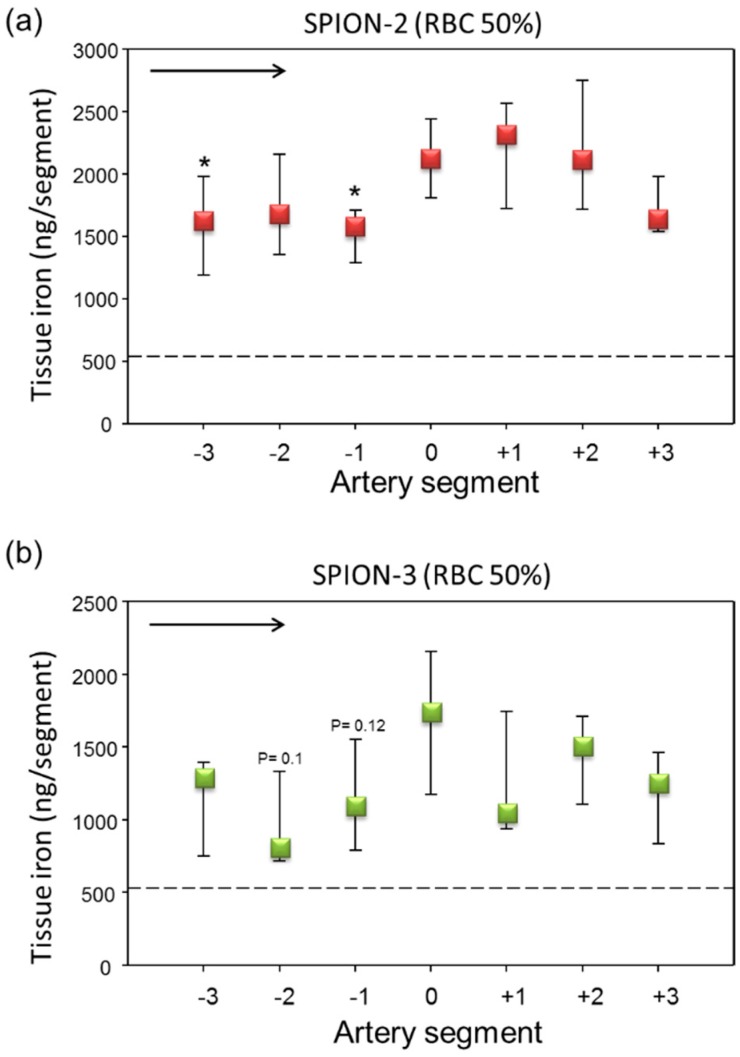
Magnetic targeting of circulating SPION-2 and SPION-3 in RBC suspensions at 50% Ht. (**a**) SPION-2 and (**b**) SPION-3 suspensions at 30 µg Fe/mL were circulated in the model for 30 min under external magnetic force positioned at segment 0. Arrow indicates the direction of flow. Shown is iron concentration of respective segments (median with 75th and 25th percentile of *n* = 5 experiments). Average control values (SPIONs suspensions without magnet) are indicated by dashed line. * *P* < 0.05 versus segment 0; trend *P* values are indicated.

**Figure 7:**
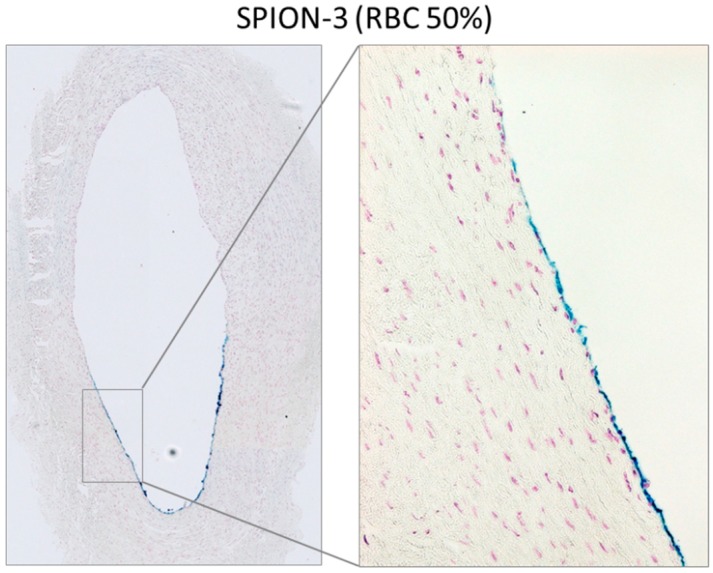
Detection of SPION-3 accumulation at the arterial wall using Prussian blue stain. Magnetically targeted segment 0 is shown as overview image and in 20x magnification for SPION-3 in RBC suspension at 50% Ht.

**Figure A2:**
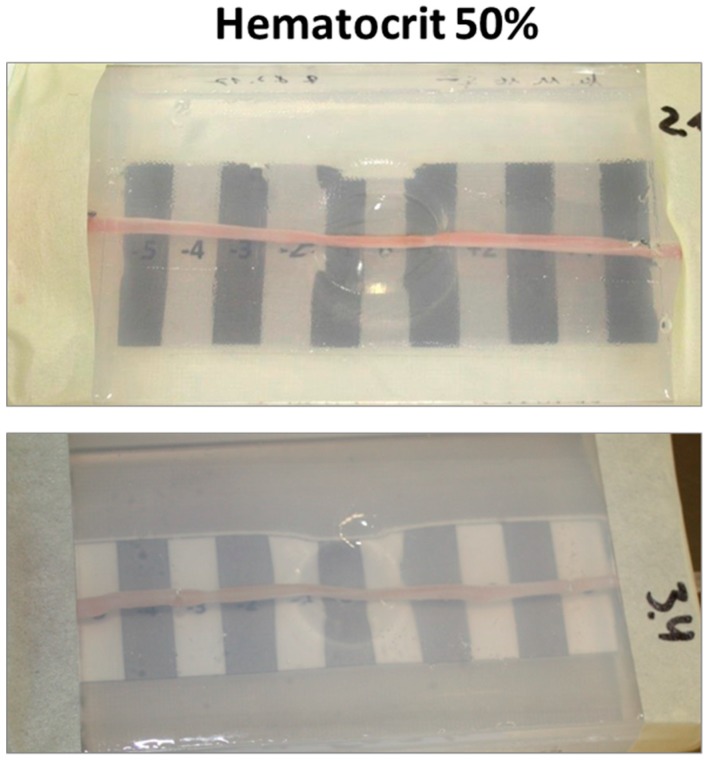
Representative macroscopic images of the artery models after the experiments with red blood cell (RBC) suspensions at 50% Ht.

When SPIONs were perfused through the artery model in RBC suspensions at 50% Ht, overall much less RBC accumulation in the artery was observed (Figure A2) as compared with whole blood. In comparison to serum, increased accumulation of iron was observed in arterial tissue. In SPION-2 samples, a shift of the peak of accumulated iron towards distal segments (0, +1, +2) was measured (Figure 6a). Interestingly, although in our previous study, we did not observe an accumulation of circulating SPION-3 suspended in cell culture media under the tip of the magnet, in the presence of RBCs, SPION-3 were successfully accumulated at the same value of magnetic field gradient (Figure 6b). The magnetic capture was sufficiently strong for clear iron visualization using histochemical staining (Figure 7).

## 3. Discussion

In order to design safe intravascular drug delivery nanosystems, their basic physicochemical characteristics and biological effects on the vascular and blood cells must be investigated [31,32]. Furthermore, the effects of hemodynamic forces on nanoparticle behavior in circulation and their adhesion to the endothelium must be considered [26]. For the delivery of nanocarriers to the vascular wall, passive and active targeting have been used. Passive targeting exploits the enhanced endothelial permeability in inflammatory diseases and cancer, which facilitates the extravasation of nano-sized particles [33]. Nanoparticles that prolong the circulation half-life of the carried drugs are therefore expected to increase the payload of drugs reaching the target-site. However, despite the presence of leaky vessels in tumors, the efficacy of drugs and passively-targeted drug carriers applied via intravenous route is often insufficient for a meaningful clinical improvement [34]. For this reason, nanoparticulate carriers that can be used for targeted drug delivery to the vascular wall constitute an attractive alternative. The active targeting of nanoparticles can be achieved by grafting the surface or the shell of the nanocarriers with specific ligands or antibodies to molecules expressed on the endothelium. However, this approach does not necessarily improve efficacy, as few nano-sized particles are expected to marginate towards human artery walls due to their small size and the specific hemodynamic conditions. MDT thus seems to represent a promising strategy of drug delivery, which results in increased drug payloads in the target tissue, at the same time reducing their systemic dose and toxicity [22,35]. Using this technique, we have recently demonstrated the possibility of SPION accumulation in abdominal aorta of living rabbits [21].

Our former study highlighted the importance of nanoparticle coating on the magnetic accumulation under arterial flow. When nanoparticles were suspended in low-serum endothelial cell culture medium, SPION-1 with lauric acid shell had the largest capacity to accumulate at the specific artery segment. SPION-2 (lauric acid/albumin-coated) were also successfully targeted, although the iron content was smaller than for SPION-1. In that study, we did not achieve magnetic accumulation of dextran-coated SPION-3 suspended in cell culture medium, likely due to the steric repulsion resulting from their specific surface chemistry [36], which may reduce the dipole–dipole interactions between the particles. Furthermore, the use of cell culture media instead of blood or human plasma for preparing the circulating SPION suspensions was a major limitation that could greatly impact the magnetic capture efficacy. Our present study was thus dedicated to investigating the effects of blood components on the accumulation of SPIONs under an external magnetic field.

**Figure A3:**
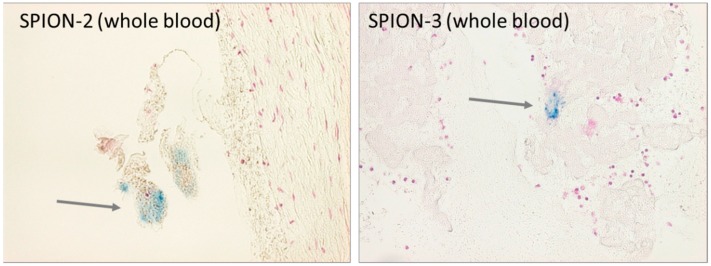
SPION entrapment in the clot formed in the artery lumen. Histological evaluation of example experiments with whole blood is shown in 20× magnification. Iron was detected using Prussian blue stain.

In the first attempts, we prepared the respective SPION suspensions using freshly isolated human plasma or whole blood. For SPION-2 samples in plasma, a strong peak in iron concentration shifted towards segment −1 was observed, whereas a milder peak with stronger variability between samples was noted for SPION-3. In the whole blood samples, a strong increase in iron content measured by AES was observed for both particle types, which may be related to partial hemolysis of the RBCs during the multiple circulation rounds within the flow system, but also to the accumulation of RBC aggregates in the artery despite stringent washing. Perfusion of plasma and whole blood suspensions through the isolated umbilical artery from a different donor resulted in a strong activation of clotting factors. This was an unexpected finding, which can however be explained by an endogenous thrombin generation in plasma, that occurs e.g., by contact with cells expressing tissue factor (TF). Thrombin is the central coagulation protease that activates clotting proteins, triggers platelet aggregation, and converts fibrinogen to fibrin [37]. Upon the contact of flowing exogenous plasma with arterial endothelial cells expressing TF, a pro-coagulative effect is induced, whereby fibrinogen contained in plasma is converted into a fibrin mesh that can affect the flow and/or entrap the flowing particles (Figure A3). This pro-coagulative effect has been previously observed in transfusion medicine, where RBC-derived microparticles isolated from blood units, or graft products for peripheral blood stem cell transplantation, were reported to initiate and propagate thrombin generation [38,39]. The use of RBCs stored for more than 28 days has been associated with an increased incidence of deep vein thrombosis in the recipient, most likely due to coagulation activation [40,41]. It must also be noted that after blood sample centrifugation, remnant platelets may remain suspended in plasma and become activated by thrombin. Our finding is of technical importance for the investigations involving human plasma and whole blood in the artery models mimicking in vivo situation. The clotting effect was not reported in the previously published studies with whole blood circulating in plastic chambers [11,12] and was not observed in our samples prior to their perfusion through the artery.

To overcome this problem, in the second set of experiments, we have therefore used human serum (cell-free part of blood devoid of clotting factors such as fibrinogen) or washed RBC resuspended in PBS to 50% Ht. Under these conditions, no clotting activation was observed in the perfused samples, either controls or nanoparticle-containing suspensions. In the serum samples, the accumulation of particles was low as compared with plasma. Slight, but not significant, peaks in iron concentration were observed around the artery segments from −1 to +1. The most notable finding refers to the results obtained with SPION-3 suspended in RBC at Ht of 50%. In our previous study, using the particle suspensions in cell culture media, we did not achieve an accumulation of circulating SPION-3 under the tip of the magnet. Here, in the presence of RBCs, SPION-3 were successfully accumulated in the target region. This finding warrants further studies, both in vitro and numerical simulations, concerning the potential interactions of SPION-3 with flowing RBCs. In this context, former in silico mathematical simulations based on the tubular vessel models predicted that magnetic accumulation of nanoparticles against the hydrodynamic drag force is not possible [42]. However, more recent simulations and experiments suggest that in situ reversible aggregates which are formed by interaction of SPIONs’ magnetic dipoles greatly contribute to magnetic accumulation of SPIONs [43,44,45]. Thus far, we have little data on how the SPION-3 coating is affected in the presence of blood proteins. In our former in vitro investigations, we did not detect the formation of hard protein corona on SPION-3, likely due to the presence of stable crosslinked dextran coating, which is nearly neutral. Slightly more protein adsorption was observed on SPION-2 (unpublished observations). However, it cannot be excluded that a lose protein corona forming on the SPION-3 may enhance the dipole-dipole interactions between the particles. Furthermore, the hemodynamic effects of blood-borne cells must be taken into account. In the arteries, characterized by large diameter and increased shear stress, the multi-file flow of RBC creates a cell-free layer at the vessel wall. As a consequence, two scenarios are possible: the particles may become pushed out of the lumen center by the forming rouleaux, thus increasing their margination, or they may be captured among the vortex-like flow among RBCs in the center of the lumen. Thus far, multi-file flow was assumed to improve margination of microparticles, and not nanoparticles, but these investigations were done in the absence of external forces. SPION-3, having a high aspect ratio and irregular branched shape may be prone to escape from the central column of RBC flow, if an external magnetic force of sufficient strength is present. Further investigations will be necessary to clarify the mechanisms of margination and magnetic accumulation of SPION-3 under arterial flow conditions.

Our study has several limitations. One of them is related to a relatively small sample size, which was limited by the availability of human umbilical arteries and resulted in low statistical power. As also explained in the methods below, with some of the samples, we could not complete the experiments, due to the variations in material quality, or postpartum blood collection procedures. Despite this fact, 64 magnetic targeting experiments were successfully performed, whereby 5 independent artery samples from different donors used for iron content measurement and 3 for histological evaluation, for each SPION type and each experimental condition. Another limitation of our model is that the short duration of the flow experiment does not allow reaching conclusions about cellular uptake of SPIONs. The 30 min experiment, after which the arterial tissue is fixed, is too short to detect particle uptake in specific cell types. We can solely observe whether the particles have tendency to accumulate at the lumen surface, or whether they penetrate vessel wall during this period of time.

Due to a major problem arising from inadequate doses of the approved nanomedicinal products reaching the diseased tissues [34], improving the targeting efficacy using external magnetic field and drug-loaded SPIONs is a very important first step in local drug delivery [46]. Our studies aim to evaluate the possibility of improved SPION accumulation and determine the best conditions for the two model particles. The need to assess the subsequent particle biodistribution, tissue kinetics and clearance, as well as immune responses is unquestionable and must be addressed in the future, but this type of analyses can only be done in a living organism, using appropriate animal models.

## 4. Materials and Methods

### 4.1. Materials

Lauric acid, epichlorohydrin, iron (III) chloride hexahydrate and dextran T40 (Mw = 40 kDa) were from Sigma Aldrich (Munich, Germany). Bovine serum albumin (BSA) and iron (II) chloride tetrahydrate were from Merck (Darmstadt, Germany). NaOH, HCl (25%), NH_3_ (25%), nitric acid (65%w/w), potassium ferrocyanide, and agarose were from Roth (Karlsruhe, Germany). All compounds used were of pharmaceutical (Ph. Eur) or highly pure (⩾99%) grade and were used without any further purification. Ringer solution was obtained from Fresenius Kabi (Bad Homburg, Germany).

### 4.2. Nanoparticle Synthesis and Characterization

The tested types of SPIONs were synthesized at the Section of Experimental Oncology and Nanomedicine, University Hospital Erlangen [27].

SPION-2: Lauric acid/BSA-coated iron oxide nanoparticles were synthesized by co-precipitation, subsequent in situ coating with lauric acid, and formation of an artificial albumin corona as described by Zaloga et al. [47]. Briefly, Fe (II) and Fe (III) salts at a defined molar ratio (Fe^3+^/Fe^2+^ = 2) were dissolved in water and stirred at 80 °C under argon atmosphere, followed by addition of NH_3_ solution (25%). The solution was heated to 90 °C and lauric acid, dissolved in acetone, was added. The brownish suspension was left to homogenate for 30 min at 90 °C. The suspension was then dialyzed multiple times against ultrapure water. Subsequently, SPIONs were stabilized by incubation with a freshly prepared 20% BSA solution, purified by centrifugal ultrafiltration (molecular weight cut-off 100 kDa), and sterilized by filtration through a 0.22 μm filter.

SPION-3: For the preparation of dextran-coated iron oxide nanoparticles, the synthesis method described by Unterweger et al. was used [48]. Briefly, Fe (II) and Fe (III) salts in molar ratios (Fe^3+^/Fe^2+^ = 2) as well as dextran T40 were dissolved in water. After cooling to 4 °C under continuous stirring and argon atmosphere, ice-cold 25% NH_3_ was added. After 5 min, the reaction mixture was heated and kept at 75 °C for a further 40 min, followed by cooling to RT and dialysis (molecular weight cut-off 8 kDa). The mixture was then cleared from excess dextran and concentrated using ultrafiltration (molecular weight cut-off 100 kDa). To stabilize the dextran coating, crosslinking was performed by adding epichlorohydrine dropwise to the nanoparticle suspension after alkalization with NaOH under vigorous stirring. The solution was then dialyzed against water, concentrated by ultrafiltration and sterile filtered through 0.22 µm membrane.

The extensive physicochemical characterization of these particles was reported in the previous publications [49,50].

### 4.3. Blood Collection

Peripheral venous blood from healthy donors was drawn into sodium citrate Monovette (106 mM, 1:10 *v/v*), or serum Monovette (Sarstedt, Germany). Citrated blood was divided into aliquots and used for flow experiment, or centrifuged at 190 g for 10 min to obtain RBCs suspension and plasma. RBCs were washed twice in PBS to remove the traces of plasma and resuspended in PBS at Ht of 50%. Serum was obtained after clotting of blood by centrifugation at 1500 g for 10 min and used for the experiments immediately. The remaining volumes of serum were stored at −80 °C for the future use. The use of human blood was approved by the local ethics committee at the University Hospital Erlangen (review No. 4449).

### 4.4. Umbilical Artery Preparation

Human umbilical arteries were isolated from freshly collected umbilical cords (kindly provided by Prof. Beckmann, Dept. of Gynecology, Universitätsklinikum Erlangen). Within 1–4 days post-partum, the cords were used for isolation of primary HUVECs from umbilical vein. After HUVECs isolation was completed, the umbilical arteries were prepared from the cords, washed and cut in 13 cm fragments, used for the flow experiment as reported previously [27]. The study was conducted according to the Declaration of Helsinki and the relevant national guidelines. The use of human material was approved by the local ethics committee at the University Hospital Erlangen (review No. 4449). Written informed consent was obtained from donors.

### 4.5. Flow Experiments

At each end of a 13 cm long artery fragment, plastic Luer connectors (Novodirect, Kehl, Germany) were fixed with surgical thread. After rinsing multiple times with Ringer solution to remove the remaining blood clots, artery fragments were placed in a plastic container and subsequently filled with pre-warmed endothelial cell culture medium to prevent the lumen collapse. Arteries were then embedded in agarose gel and connected to the peristaltic pump (model ISM 915, Ismatec, Wertheim, Germany) and SPION reservoir using Ismaprene tubes (Ismatec). In this set-up, samples of human plasma, serum, whole blood and washed red blood cells (50% Hct) containing SPIONs (30 µg/mL) were perfused through the artery with or without an external magnetic force for 30 min, at the flow rate of 4.8 mL/min (corresponding to the shear stress of 5 dyne/cm^2^). The selected concentration of circulating SPIONs (30 µg/mL) was comparable to the maximal systemic dose of iron oxide-based contrast agent ferumoxtran (2.6 mg/kg body weight, corresponding to 33 µg/mL blood in humans). In the experiments with magnetic field gradient (Siemens electromagnet, Siemens AG Erlangen, Germany), the magnetic field gradient at the tip of the pole shoe was set for 72 T/m, which corresponded to about 40 T/m field gradient at the center of artery lumen. The tip of the magnet was positioned directly on top of the agarose gel surface, in a 5 mm distance from the wall of embedded artery. As controls, SPIONs without external magnetic field gradient were used. After the experiment, the arteries were rinsed with Ringer solution for 2 min, or for 15 min in the case of RBC-containing suspensions. Subsequently, the models were disconnected from the pump and arteries were cut into segments of 1 cm length, numbered (−5, . . . ,0, . . . ,+5) from in-flow to out-flow, followed by additional rinsing of single sections with Ringer solution. Due to the variations in material quality, postpartum blood collection procedures and other independent factors, some artery samples were not suitable for performing the experiment, especially if the artery was punctured. In total, we collected above 80 artery samples for the study, and with 64 of them we successfully completed the magnetic targeting experiments. For each SPION type and each experimental condition, 5 independent artery samples from different donors were used for subsequent iron content measurement and 3 for histological evaluation. Additionally, *n* = 2–4 samples were used as negative controls (with SPIONs in respective media, without magnet). Due to the fact that the inflow and outflow regions (−5, −4 and +4, +5) are prone to a processing damage during the placement of connectors, which may lead to an aberrant accumulation of circulating SPIONs at the injured region [27], only the regions from −3 to +3, which experience uniform flow conditions, were considered in the analyses.

### 4.6. Iron Content Measurement with AES

The tissue iron concentration was quantified with microwave plasma atomic emission spectroscopy (MP-AES, 4200 device, Agilent, Waldbronn, Germany). Following the flow experiment, the rinsed tissue segments were dried for 2 h at 90 °C, dissolved in 100 µL of 65% nitric acid for 10 min at 95 °C and 600 rpm using a thermomixer. After addition of 900 µL of water, the emission spectrum of the samples was analyzed and compared to the standard curves. Tissue iron concentration values were given as ng Fe per artery segment.

### 4.7. Tissue Iron Content by Histology

The tissue segments were stained with Prussian blue to assess the uptake of circulating SPIONs. Briefly, the artery segments were placed in embedding cassettes, fixed in 4% formaldehyde solution (Roth) in PBS buffer for 2 days. Afterwards, samples were dehydrated in an ascending isopropanol sequence and finally embedded in paraffin. Blocks were then cut using the microtome and the paraffin-embedded serial sections of 4-µm-thickness were dewaxed in xylene, rehydrated in ethanol, and immersed in 1:1 solution of hydrochloric acid (2%) and potassium ferrocyanide (2%) for 30 min at RT. Nuclei of the cells were counterstained with eosin, followed by rinsing with distilled water. As a mounting medium, Mowiol (Sigma-Aldrich) was added to extend staining durability. Images were taken using Axio Observer.Z1 microscope (Zeiss, Jena, Germany).

### 4.8. Statistics

The differences in magnetic accumulation of SPIONs between the different segments of the artery model were calculated after performing a normality test (Shapiro–Wilk), using t-test in samples that passed the normality test and non-parametric Mann–Whitney U-test in those, which failed the normality test. Data were expressed as median with 75th and 25th percentile, unless stated otherwise. *P* < 0.05 was considered statistically significant.

## 5. Conclusions

In conclusion, our studies identified the clotting effect induced by perfusion of plasma and whole blood in the human artery model as a potential confounding factor. The presence of serum did not greatly affect the magnetic capture of SPION-2, while in the presence of RBCs, the iron accumulation was increased, with a peak shifted to distal artery region. The presence and rheological behavior of RBCs in the arterial flow improved the capturing efficacy of SPION-3. Further investigations and numerical simulations will be necessary to clarify the mechanisms of margination and accumulation of SPION-3 under arterial flow conditions. This knowledge will be essential to ensure a broader and more efficient implementation of these particles in clinical practice in the future.

## Supplementary Materials

The following are available online at https://www.mdpi.com/1420-3049/24/14/2588/s1, Table S1: Physicochemical characterization of the tested SPIONs, Figure S1: Transmission electron microscopy (TEM) images of the tested SPIONs, Figure S2: Blood stability of the tested SPIONs, Figure S3: Effects of SPIONs on plasma coagulation.
