# Supplementary material for: Magnetic Accumulation of SPIONs under Arterial Flow Conditions: Effect of Serum and Red Blood Cells
Source: Molecules. 2019 Jul 16;24(14):2588. doi: 10.3390/molecules24142588 (PMC6681042; doi:10.3390/molecules24142588)
Supplement: Supplementary file 1 [file molecules-24-02588-s001.pdf]

## Supplementary Materials

**Hennig et al.**

**Manuscript ID: molecules-520234**

**Supplementary Table 1.** Physicochemical characterisation of the tested SPIONs.

| | **SPION-2** | **SPION-3** |
|---|---|---|
| Core size [nm] | 8.9 ± 2.2 | 4.3 ± 0.9 |
| Hydrodynamic diameter [nm] | 71 | 74 |
| ζ-potential [mV] | -18.1 | -3.8 |
| Coating | LA/BSA | crosslinked dextran T-40 |

The hydrodynamic size (Z-average size) and ζ-potential of the nanoparticles were determined with a Zetasizer Nano ZS (Malvern). BSA, bovine serum albumin; LA, lauric acid; SPIONs, superparamagnetic iron oxide nanoparticles.

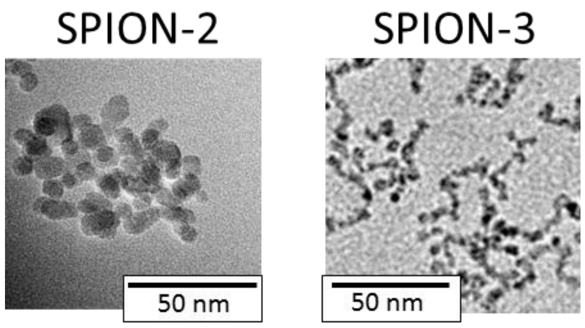


**Supplementary Figure 1**. Transmission electron microscopy (TEM) images of the tested SPIONs [1,2].

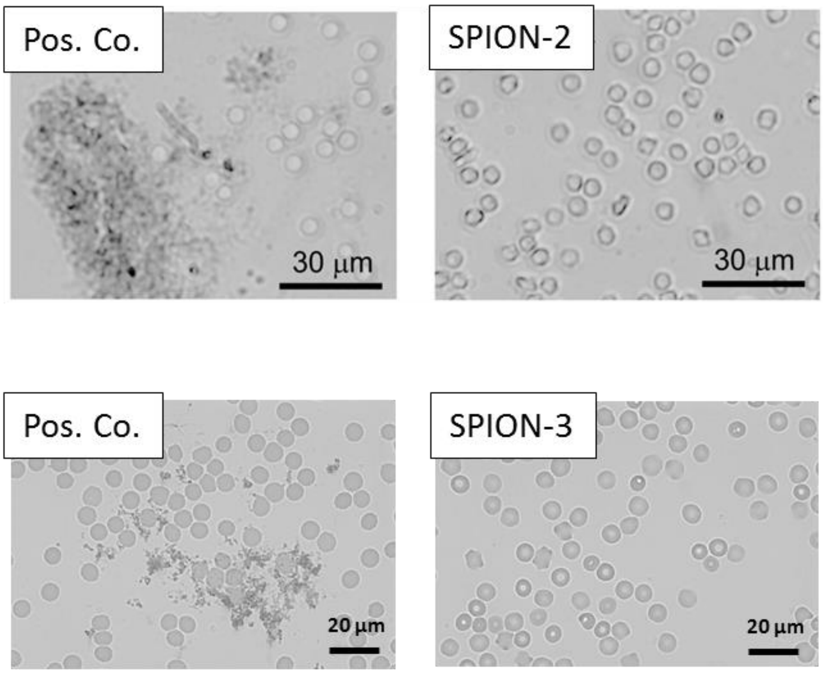


**Supplementary Figure 2**. Blood stability of the tested SPIONs. Blood stability was investigated in EDTA-anticoagulated sheep (SPION-2 [3]) or rabbit (SPION-3 [2]) whole blood. Blood sample was mixed with SPIONs to an iron concentration of 1 mg/mL. Lauric acid-coated SPIONs served as positive control. Microscopic images were taken after 45 min incubation.

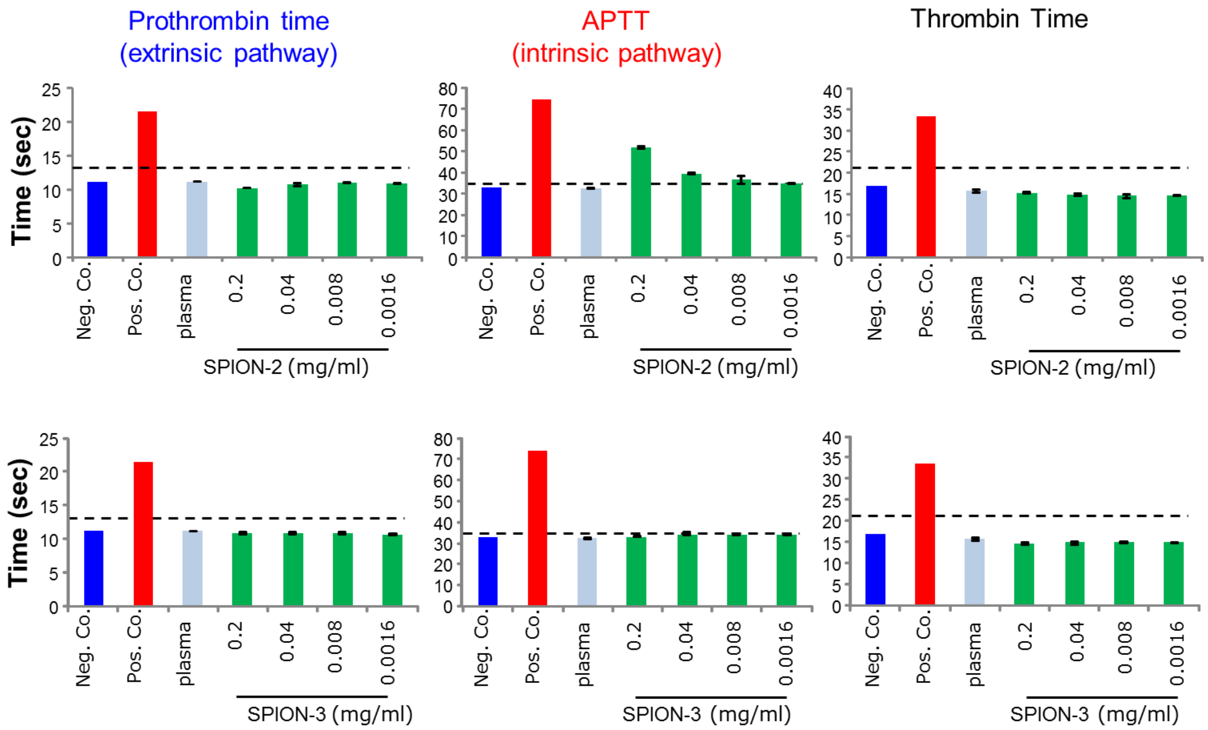


**Supplementary Figure 3**. Effects of SPIONs on plasma coagulation. Platelet poor human plasma pooled from 3 donors was treated with SPIONs for 30 minutes. Afterwards, the respective coagulation activation reagent was added to each sample (Neoplastine for prothrombin time, $CaCl_2$ for activated partial thromboplastin time (APTT), or thrombin for thrombin time) and their respective coagulation time was measured. Means ± SD of replicate samples are shown. Upper panel: SPION-2 (data courtesy of Dr. Christina Janko, SEON, University Hospital Erlangen); Lower panel: SPION-3 [2].

## References


1. Janikowska, A.; Matuszak, J.; Lyer, S.; Schreiber, E.; Unterweger, H.; Zaloga, J.; Groll, J.; Alexiou, C.; Cicha, I. A novel human artery model to assess the magnetic accumulation of SPIONs under flow conditions. *Sci Rep* **2017**, *7*, 42314, doi:10.1038/srep42314.
2. Unterweger, H.; Janko, C.; Schwarz, M.; Dezsi, L.; Urbanics, R.; Matuszak, J.; Orfi, E.; Fulop, T.; Bauerle, T.; Szebeni, J., et al. Non-immunogenic dextran-coated superparamagnetic iron oxide nanoparticles: a biocompatible, size-tunable contrast agent for magnetic resonance imaging. *Int J Nanomedicine* **2017**, *12*, 5223-5238, doi:10.2147/IJN.S138108.
3. Friedrich, R.P.; Janko, C.; Poettler, M.; Tripal, P.; Zaloga, J.; Cicha, I.; Durr, S.; Nowak, J.; Odenbach, S.; Slabu, I., et al. Flow cytometry for intracellular SPION quantification: specificity and sensitivity in comparison with spectroscopic methods. *International Journal of Nanomedicine* **2015**, *10*, 4185-4201, doi:10.2147/Ijn.S82714.
